# Depression among caregivers of cancer patients: Updated systematic review and meta‐analysis

**DOI:** 10.1002/pon.6045

**Published:** 2022-10-17

**Authors:** Asres Bedaso, Getiye Dejenu, Bereket Duko

**Affiliations:** ^1^ Hawassa University College of Medicine and Health Sciences School of Nursing Hawassa Ethiopia; ^2^ Australian Centre for Public and Population Health Research School of Public Health Faculty of Health University of Technology Sydney Ultimo New South Wales Australia; ^3^ Curtin School of Population Health Faculty of Health Sciences Curtin University Perth Western Australia Australia

**Keywords:** cancer, cancer patient, caregivers, depression, family, meta‐analysis, oncology, prevalence, Spouse, systematic review

## Abstract

**Background:**

It is imperative to provide care for patients with terminal illnesses such as cancer, though it demands time, financial resources and other unmet needs. Subsequently, caregivers might be exposed to psychological stress and other mental health problems. Previous meta‐analysis finding shows caregivers of cancer patient suffer from depression. During the past 4 years, there has been a considerable increase in the number of newly studies, and we therefore intended to update this finding and provide current global prevalence of depression among caregivers of Cancer patients.

**Methods:**

We searched PubMed, SCOPUS, CINAHIL, Embase, and PsychINFO to identify peer‐reviewed studies which reported the prevalence of depression among caregivers of cancer patients using pre‐defined eligibility criteria. Studies were pooled to estimate the global prevalence of depression using a random‐effect meta‐analysis model. Heterogeneity was assessed using Cochran's Q and *I* ^2^ statistics. Funnel plot asymmetry and Egger's regression tests were used to check for publication bias.

**Result:**

Our search identified 4375 studies, of which 35 studies with 11,396 participants were included in the meta‐analysis. In the current review, the pooled prevalence of depression among caregivers of Cancer patients was 42.08% (95% CI: 34.71–49.45). The pooled prevalence of depression was higher in the studies that used cross‐sectional data (42%, 95% CI: 31–52) than longitudinal data (34%, 95% CI: 18–50). We also observed a higher rate of depression among female caregivers when compared to their male counterparts (57.6%) (95% CI: 29.5–81.5).

**Conclusion:**

Globally, around two in five cancer patient caregivers screened positive for depression, which needs due attention. Routine screening of depressive symptoms and providing psychosocial support for caregivers is crucial.

## 1 BACKGROUND

Globally, Cancer is the leading cause of mortality, accounting for approximately 10 million deaths in 2020.^1^ Caregivers of cancer patients play an essential role in reducing mortality of cancer patients through effective palliative care and supporting clinical management.^2^ More than 90% of individuals living with Cancer have a caregiver (a friend or family member) who can provide care throughout their disease and treatment.^3^ Caregivers play a leading role in community‐based models of cancer care,^4^ and their position can extend for several years.^5^

Even though some caregivers recognize their role positively,^6^ the health impact of caring is substantial.^7^ But some caregivers consider the role as an obligation, perceiving they don't have other options.^2^ Despite caregivers' critical but demanding role, they are not getting enough support.^4^, ^8^, ^9^ As a result, caregivers experience unmet needs,^10^ leading to psychological distress.^11^ Besides, the diagnosis of Cancer itself can also have an adverse impact on the mental health of a family caregiver.^12^ Caregivers of terminal cancer patients may suffer an even higher burden as the patient's health condition deteriorates, leading caregivers to be physically and emotionally exhausted.^13^ Studies demonstrate that the caregiver's burden might increase because of the unmet need of the patient.^14^ The burden caregivers of cancer patients experience is strongly linked with the patient's well‐being.^15^ If the unmet needs of caregivers of cancer patients are not addressed, it will affect the psychological well‐being of caregivers^16^ and the health outcomes of cancer patients.^17^

Epidemiological studies reported a higher rate of depression (12%–59%)^18^ and anxiety (30%–50%)^15^, ^18^ among caregivers of cancer patients. A study conducted on family caregivers of home‐based palliative care patients (*n* = 300), found that around half of the caregivers (50%) met the criteria for psychological distress.^19^ A global level systematic review and meta‐analysis reported a pooled prevalence of 42.3% depression and 46.55% anxiety among caregivers of Cancer patients.^20^ Surprisingly, this high burden of mental health problems among caregivers didn't get attention and is subsequently not yet addressed clinically.^21^

Caregivers' mental health can be enhanced by providing routine psychosocial support.^22^, ^23^, ^24^ Further, health policymakers should develop evidence‐based interventions capable of improving caregivers' physical and psychological well‐being.^25^, ^26^ However, updating data on the burden of Caregivers' mental health problems should be prioritised before designing interventional strategies.^26^

The availability of updated evidence on the burden of depression can help synthesize updated knowledge on the topic and address the need to develop policies and strengthen programs for caregivers of cancer patients. Despite variations in the magnitude of depression among caregivers across different countries, pooling the available up‐to‐date evidence and reporting the prevalence more precisely might help policymakers prioritize the problem and encourage governments to act accordingly. A systematic review and meta‐analysis on a similar topic by Geng et al. was published in 2018 and summarized 17 studies through March 2018, reporting a 42.3% pooled prevalence of depression.^20^ During the past 4 years, there has been a considerable increase in the number of available data, and we therefore intended to update this finding and provide current global prevalence of depression among caregivers of Cancer patients.

## 2 METHODS

### 2.1 Search strategy and selection process

A systemic review and meta‐analysis was conducted using studies that examined the prevalence of depression among caregivers of Cancer patients. The strategy for literature search, selection of studies, data extraction, and reporting of results for the current review was designed following the PRISMA (Preferred Reporting Items for Systematic Reviews and Meta‐Analyses) guidelines^27^ (Supplementary File S1). The protocol for the current review has been registered in PROSPERO (CRD42022297623).

Five electronic databases (PubMed, SCOPUS, CINAHIL, Embase, and PsychINFO) were searched to identify studies that report the prevalence of depression among caregivers of Cancer patients. Searching in PubMed was performed using the following terms: (Prevalence OR Magnitude OR Epidemiology OR Incidence OR Estimates OR Burden) AND (depression OR depressive symptoms OR depressive disorder OR major depressive disorder OR major depression) AND (caregivers of cancer patient OR carers of cancer patients OR family of cancer patient OR friend of Cancer patient OR partner of cancer patient OR Spouse of cancer patient). Database‐specific subject headings associated with the above terms were used to screen studies indexed in SCOPUS, CINAHIL, Embase, and PsychINFO databases. Besides, we observed the reference lists of published studies to identify additional articles.

### 2.2 Eligibility criteria

In the current review, we have included observational studies reporting the prevalence of depression among caregivers of Cancer patients and published in English language. Eligible studies included for this review had to fulfil the following criteria: first, the type of study has to be observational (cross‐sectional, nested case‐control, or follow‐up studies). Follow‐up studies are longitudinal studies that employed continuous or repeated measures to follow individual Caregivers of cancer patients over time. Second, the study participants should be adults (whose age is greater than or equal to 18 years). Third, the measurement of depression has to be a diagnostic or validated screening tool.

Studies that reported the pooled prevalence of depression, RCTs (Randomised Controlled Trials) had a poor quality score on the New Castle Ottawa Scale (NOS), duplicate studies, conference proceedings, commentaries, abstracts, reports, short communications and letters to editors were excluded.

### 2.3 Data extraction and study quality assessment

Data were extracted using a specific form developed by authors. The data extraction form included the following information: Name of the author, year of publication, country, study design, sample size, tools used to assess depression, the number of positive cases for depression, the prevalence of depression, stage of cancer, a primary site of cancer, and the relationship of caregivers with the patient. AB conducted the primary data extraction, and then BD assessed the extracted data independently. Any disagreements and discrepancies were resolved through discussion with the third author GD.

The methodological qualities of each included article were assessed by using a modified version of the Newcastle‐Ottawa Scale.^28^ The methodological quality and eligibility of the identified articles were evaluated by two reviewers (AB and GD), and disagreements among reviewers were resolved through discussion with the third Author (BD). Finally, studies with a scale of ≥5 out of 10 were included in the current meta‐analysis.

### 2.4 Data analysis

The prevalence report extracted from all the included primary studies were meta‐analyzed. We have examined publication bias by visual inspection of a funnel plot and Egger's regression test.^29^, ^30^ A *p*‐value <0.05 was used to declare the statistical significance of publication bias. Studies were pooled to estimate pooled prevalence and 95% CI using a random‐effect model.^31^ We have assessed heterogeneity using Cochran's Q and *I* ^2^ statistics.^32^ *I* ^2^ statistics is used to quantify the percentage of the total variation in the study estimate due to heterogeneity. Due to the presence of significant heterogeneity, we have conducted a subgroup analysis based on the study design, gender of participants, and the type of tool used to detect depression. All the extracted data were analyzed using STATA 16.

## 3 RESULTS

### 3.1 Identification of studies

**FIGURE 1:**
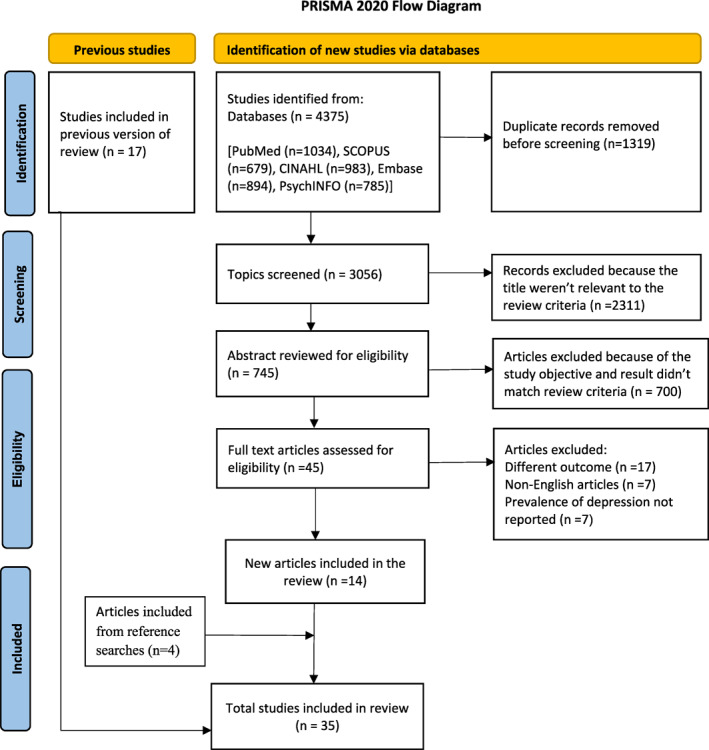
PRISMA flow chart of the study identification process for the systematic reviews and meta‐analysis

We retrieved a total of 4375 records via bibliographic databases searching. After removing duplicates, 3056 records were contained for title screening. Then, after removing 2311 records because of the irrelevance of the topic to the review criteria, 745 studies were retrieved for abstract screening. Seven hundred articles were excluded during the abstract screening because of the discrepancy between the study results and our review objective. Subsequently, the remaining 45 full‐text articles were assessed for eligibility. Of these screened full‐text articles (*n* = 45), 14 new articles, together with four articles identified through reference searching fulfilled the criteria for our meta‐analysis. Then, together with 17 studies from the previous review,^16^ a total of 35 articles were included in the current updated systematic review and meta‐analysis (Figure 1).

### 3.2 Characteristics of included studies

Table 1 has summarized the key characteristics of the studies included in this systematic review and meta‐analysis. The sample size of study participants who completed a study ranged from 41^33^ up to 2743 participants.^34^ The studies included in this review were available online between the year 1998^35^ and 2018.^36^ Seven studies were conducted using a prospective longitudinal study design^37^, ^38^, ^39^, ^40^, ^41^, ^42^, ^43^ whereas the remaining twenty eight studies used a cross‐sectional study design to examine depression among caregivers of cancer patients.

**TABLE 1:** Characteristics of the studies included in this systematic review and meta‐analysis

First author, year	Country	Sample size	Study design	The primary site of cancer	Stage of cancer	Relationship to the patient	The tool used to assess depression/Cut‐off score	Prevalence of depression (case/total sample) (%)	NOS score
Given, 2005^56^	USA	152	Longitudinal	Breast, colon, lung and prostate cancer	I‐IV	Spouse, Daughter/Son or others	CESD‐20 (≥16 out of 60)	47/152 = 31%	8
Young, 2008^57^	South Korea	310	CS	Stomach, liver, lung, colorectal, uterus, breast	I‐IV	Spouse, family member, or friend	BDI (>13 out of 63)	207/310 = 66.7%	8
Tang, 2007^58^	Taiwan	170	CS	Any type	IV	Spouse or other family member	CESD‐20 (≥16 out of 60)	75.90%	8
Grov, 2005^47^	Norway	96	CS	GI, Prostate, breast and others	IV	Spouse or other family member	HADS‐D (>8 out of 21)	23/96 = 24%	8
Lauren, 2005^38^	USA	200	Longitudinal	Any type	IV	Spouse, child, sibling, friend, parent, other family member	DSM IV	9/200 = 4.5%	8
Mohammed 2012^48^	Iran	63	CS	Breast cancer	I‐IV	Family member or friends	BDI (>10 out of 63)	38/63 = 60.3%	6
Jill, 2002^59^	Canada	44	CS	Colorectal, pancreatic, leukemia, stomach, lung carcinoma & others	III and IV	Spouse, daughter or son	Short	23/44 = 53%	5
Chris, 1999^39^	Netherlands	148	Longitudinal	Colorectal carcinoma	I‐IV	Spouse	Form of the profile of mood states	71/148 = 48%	7
Serge, 2006^40^	Canada	212	Longitudinal	GI, lung and others	I‐IV	Spouse/other family member/friend	CES‐D 20 (≥16 out of 60)	41/212 = 19.2%	8
Patricia, 2000^44^	USA	51	CS	Lung, colorectal, or other sites	III and IV	Spouse, adult child and other family member	IDPESQ	27/51 = 52.9%	5
Mariah, 2006^60^	South Korea	103	CS	Gastric cancer	III and IV	Family member	CES‐D 20 (≥16 out of 60)	56/103 = 55%	6
Price, 2010^41^	Australia	373	Longitudinal	Ovarian cancer	I‐IV	Spouse or other family member	CES‐D 20 (≥16 out of 60)	22/373 = 5.9%	8
Victoria, 1998^35^	USA	164	CS	Any type	I‐IV	Adult daughter	CES‐D 20 (≥16 out of 60)	49/164 = 30%	8
Jacquelyn, 2000^45^	USA	117	CS	Any type	IV	Family member	CES‐D 20 (≥16 out of 60)	58/117 = 50%	7
Paula, 2005^46^	USA	104	CS	Brain tumor	I‐IV	Spouse, other family member	CES‐D 20 (≥16 out of 60)	38/104 = 36.9%	6
Irma, 2007^33^	USA	41	CS	Brain and neck	I‐IV	Spouse, other family member	CES‐D 20 (≥16 out of 60)	8/41 = 20%	5
Zümrüt, 2012^61^	Turkey	60	CS	Any type	I‐IV	Family member	HADS‐D (>8 out of 21)	34/60 = 57%	5
Badger, 2007^42^	USA	96	Longitudinal	Breast cancer	I‐IV	Partner	CESD‐20 (>16 out of 60)	71/96 = 74%	8
Heckel, 2015^10^	Australia	150	CS	Any type	IV	Spouse/partner	CESD‐20 (≥16 out of 60)	54/150 = 36%	9
Kim, 2007^62^	USA	98	CS	Colorectal	I‐IV	Family or close friend	CES‐D 20 (≥16 out of 60)	29/98 = 29.6%	8
Tang, 2013^43^	Taiwan	193	Longitudinal	Any type	IV	Family caregiver	CES‐D 20 (≥16 out of 60)	106/193 = 55%	8
Yang, 2012^63^	China	312	CS	Any type	IV	Spousal/family caregiver	CES‐D 20 (≥16 out of 60)	198/312 = 63.5%	8
Jaafar, 2014^49^	Malaysia	130	CS	Breast cancer	I‐IV	Any family member	DASS‐21 (NA)	64/130 = 49.2%	9
Gotze, 2014^50^	Germany	106	CS	Any type	I‐IV	Family caregiver	HADS‐D (≥10)	30/106 = 28%	9
Leroy, 2015^51^	France	60	CS	Any type	I‐IV	Family caregiver	HADS‐D (≥10)	27/60 = 45%	7
Park, 2013^64^	South Korea	897	CS	Any type	I‐IV	Family caregiver	HADS‐D (≥8)	737/897 = 82.2%	9
Yu, 2017^65^	China	309	CS	Leukemia	I‐IV	Family caregiver	HADS‐D (≥8)	65/309 = 21%	8
Nielsen, 2017a^34^	Denmark	2743	CS	Any type	IV	Family caregiver	BDI‐II (21 item) (NA)	473/2743 = 17.2%	8
Nielsen, 2017b^66^	Denmark	1989	CS	Any type	IV	Family caregiver	BDI‐II (21 item) (NA)	312/1989 = 15.6%	8
Rhee, 2008^57^	South Korea	310	CS	Any type	I‐IV	Family caregiver	BDI (>13)	207/310 = 67%	7
Trevino, 2018^36^	USA	540	CS	Any type	I‐IV	Family caregiver	HADS‐D (≥10)	22/518 = 4.2%	8
Mhaidat, 2011^52^	Jordan	302	CS	Any type	I‐IV	Family caregiver	HADS‐D (≥8)	247/302 = 82%	7
Catende, 2017^53^	Uganda	119	CS	Any type	I‐IV	Family caregiver	HDS‐D (≥11)	31/119 = 26%	8
Sahadevan, 2019^54^	India	384	CS	Breast cancer	I‐IV	Family caregiver	HAM‐D (≥8)	202/384 = 53%	8
Lutfi M, 2019^55^	Iraq	250	CS	Leukemia	I‐IV	Family caregiver	BDI‐II (≥17)	180/250 = 72%	7

Abbreviations: CS, Cross‐sectional, NA, Data not available.

Of the thirty five studies included, ten studies were from USA,^33^, ^35^, ^37^, ^38^, ^44^, ^45^, ^46^ four studies from South Korea, and two studies each from Canada, Australia, Denmark, China, Taiwan and Turkey. Also, one study from each of the following countries namely, Norway,^47^ Iran,^48^ The Netherlands,^39^ Malaysia,^49^ Germany,^50^ France,^51^ Jordan,^52^ Uganda,^53^ India^54^ and Iraq.^55^

Regarding the tool used to screen/diagnose depression, nineteen studies have used the Center for Epidemiological Studies‐Depression (CES‐D‐20), eight studies used Hospital Anxiety and Depression scale (HADS‐D), four studies used the Beck Depression Inventory (BDI), one study each used ‘Indice de détresse psychologique de Santé Québec (IDPESQ)’, fourth version of the Diagnostic and Statistical Manual of Mental Disorders (DSM‐IV), Depression Anxiety Stress scale and Hamilton rating scale for depression (HAM‐D).

### 3.3 The quality of studies included in the review

The methodological quality of studies included in this review was assessed by the modified version of the Newcastle‐Ottawa Scale.^28^ Of the total thirty‐five studies checked for methodological quality, twenty‐two (64.5%), nine (19.4%), and four studies (12.9%) were of high (NOS score ≥8), moderate (NOS score 6–7) and low methodologic quality, respectively (Table 1).

### 3.4 Meta‐analysis

**FIGURE 2:**
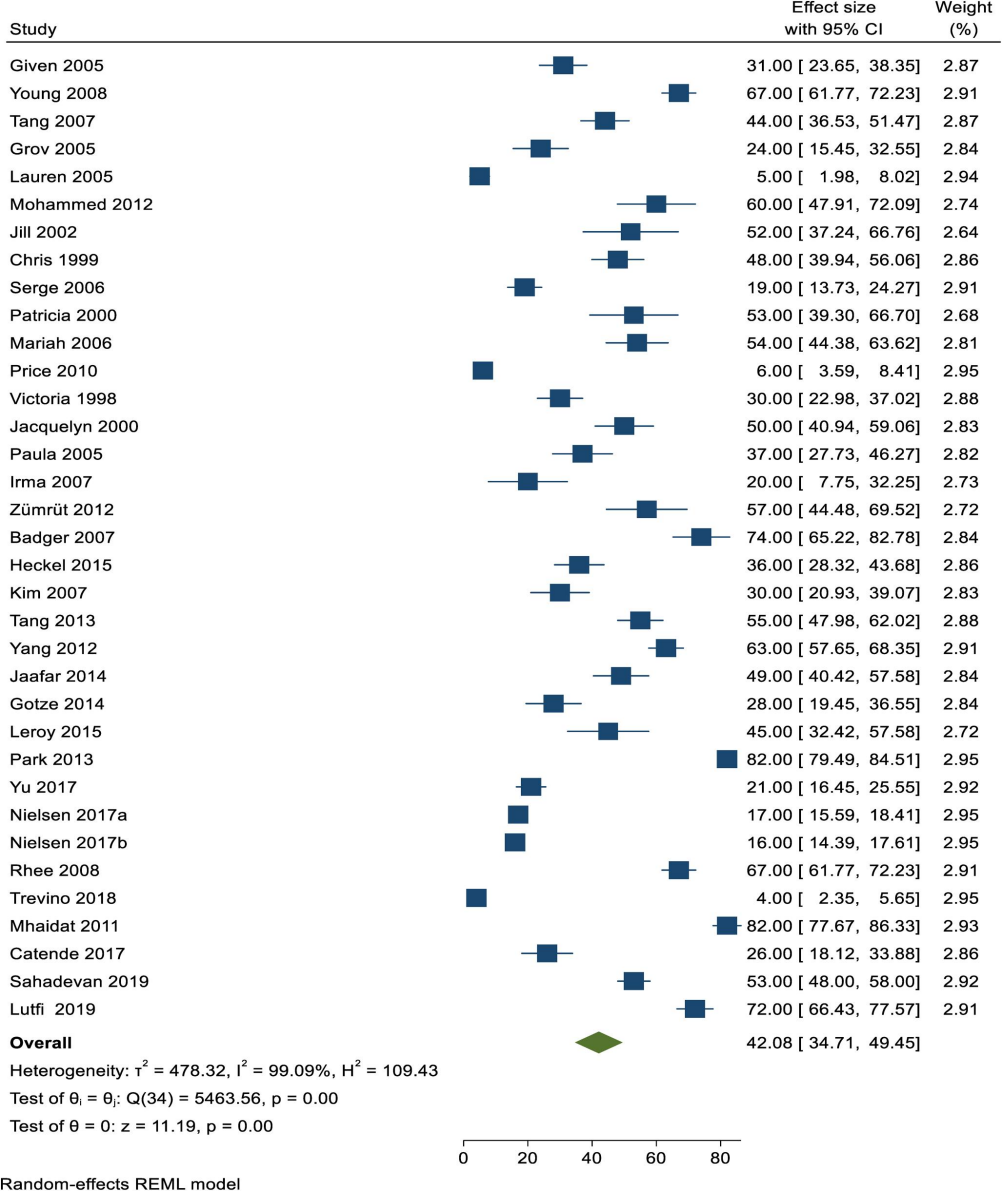
Fore plot showing the pooled prevalence of depression among caregivers of cancer patients

Globally, the pooled prevalence of depression among caregivers of Cancer patients was 42.08% (95% CI: 34.71–49.45) and the observed heterogeneity was substantial (*I* ^2^ = 99.09%, *Q* = 5463.56, *p* < 0.001) (Figure 2).

### 3.5 Subgroup analysis and publication bias

Table 2 has presented the findings of subgroup analysis. The prevalence of depression that was noted in this review was diverse by the methodological designs the studies followed, the tools the studies used to measure depression, the stages of cancer, and the gender of participants.

**TABLE 2:** Subgroup analysis for the prevalence of depression among caregivers of cancer patients

Subgroups	No. of studies	Prevalence (%)	95% CI	Heterogeneity within the study (*I* ^2^ statistics (%))
Types of tools used to assess depression				
BDI	4	65.3	58.5–72.0	63.7
CES‐D	19	42.5	31.9–53.09	97.8
HADS‐D	8	34.4	21.0–47.8	99.5
IDPESQ	1	19	13.7–24.3	0.00
DSM‐IV	1	5	1.98–8.02	0.00
DASS‐21	1	82	79.4–84.5	0.00
HAM‐D	1	53%	48.0–47.9	0.00
Study design used				
Cross‐sectional	28	42.2	33.8–54.6	99.4
Longitudinal	7	33.6	17.7–49.6	98.7
Gender of study participants				
Male	4	34.4	12.4–66.1	97.4
Female	4	57.6	29.5–81.5	90.03
Stage of cancer				
Terminal stage (stage 3 and 4)	12	38.64	27.67–49.62	98.97
Others	23	43.82	34.10–53.54	98.93

The pooled prevalence of depression among caregivers of cancer patients differed when the methodological designs of the studies varied. For example, the pooled prevalence of depression in the studies that used a cross‐sectional study design (42.2%) was higher than those studies that have followed a longitudinal study design (33.6%).

Further, the higher prevalence of depression was observed in the studies that used DASS‐21 (82%), followed by BDI (65.3%), and HAM‐D (53%), whereas the lowest pooled prevalence was observed in the study that used DSM‐IV (5%). We further employed a subgroup analysis limiting the analysis to the studies that have reported prevalence for male and female caregivers of cancer patients. A higher prevalence of depression found among female caregivers (57.6%) than males (34.4%). Finally, a subgroup analysis was conducted based on the stages of cancer (terminally ill patient vs. stages one and two). A slightly higher prevalence of depression was observed among caregivers of stage one and stage 2 cancer patients (43.8%, 95% CI: 34.10–53.54) compared to caregivers of terminally ill cancer patients (38.64%, 95% CI: 27.67–49.62).

**FIGURE 3:**
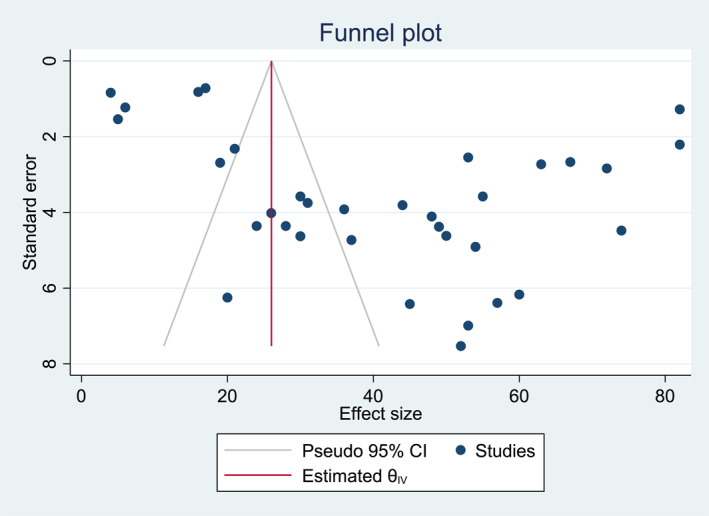
Funnel plot shows no evidence of publication bias among included studies

For the overall meta‐analysis of the prevalence of depression among caregivers of Cancer patients, both the visual inspection of the funnel plot (Figure 3), and Egger's regression, showed no evidence of publication bias (*B* = 3.05, SE = 2.086, *p* = 0.144).

## 4 DISCUSSION

Our systematic review and meta‐analysis, estimating the global level pooled prevalence of depression among caregivers of Cancer patients revealed an interesting finding. Thirty five studies, with 11,396 caregivers, conducted across eighteen countries, were included in the current review.

Overall the global prevalence of depression among caregivers of Cancer patients across studies was 42.08% (95% CI: 34.71–49.45). The pooled prevalence in the current review was comparable with a pooled prevalence reported by a previous systematic review and meta‐analysis (42.3%) conducted before 4 years.^20^ However, the pooled prevalence of depression in the current study was remarkably higher than the prevalence of depression cases among a general population reported in 2015 (9%).^67^ This high prevalence of depression among caregivers of cancer patients might be due to providing care for the patient for long hours per day without getting enough support.^4^, ^8^, ^9^ Besides, those caring for terminal cancer patients may suffer from an even higher burden as the patient's health condition deteriorates, leading caregivers to be physically and emotionally exhausted.^13^ Our review also indicated caregivers included in 12 studies (*n* = 6168) care for terminally ill cancer patients. Furthermore, the unmet need of the patient can also lead caregivers to experience psychological distress.^14^

The subgroup analysis conducted using the study design as a moderator demonstrated that the pooled prevalence of depression in the studies that used a cross‐sectional study design (42%) was higher than studies which followed a longitudinal study design (34%). The possible reason for a higher prevalence of depression among cross‐sectional studies might be due to the effect of decreasing participation rates over time on longitudinal studies.^68^ Participant non‐response is higher among the most vulnerable members of society, such as those with young age, economically poor and poor health conditions. Therefore the absence of these individuals from longitudinal studies might lower the prevalence of depression, as these individuals are most likely to experience poor mental health. Second, the sample size of the studies which employed a cross‐sectional design had 9627 caregivers of cancer patients compared to that of studies that used a longitudinal study design (*n* = 1374). Of these twenty eight cross‐sectional studies, ten had only caregivers of terminally ill cancer patients (stage III and IV) (*n* = 5775) who may suffer a higher burden as the patient's health condition deteriorates. In contrast, of the total seven studies that used a longitudinal design, only two included caregivers of terminally ill cancer patients (*n* = 393). This finding suggests the need for further studies examining depression among caregivers of cancer patients at different stages of illness with representative sample size and robust study design.

The subgroup analysis conducted using the tool employed to identify depression have showed substantial variation across studies among caregivers of cancer patients. Most importantly, the higher prevalence of depression was observed in the studies that used DASS‐21 (82%), followed by BDI (65.3%), and HAM‐D (53%), whereas the lowest pooled prevalence was observed in the study that used DSM‐IV (5%). This might be due to many studies included in the current meta‐analysis used CES‐D (i.e., 19 out of 35 studies (54.3%)) and BDI (i.e., 4 out of 31 studies (11.4%)) to examine depressive symptoms across studies, which is a highly sensitive screening tool compared to others,^69^ suggesting the possibility of overestimation of depression rate. DASS‐21 is also a screening tool for probable depressive symptoms, which is highly sensitive in case detection compared to DSM‐IV, a diagnostic tool. Besides, the observed variation might be due to the difference in the psychometric properties of measures employed across studies.

Our subgroup analysis indicated that the prevalence of depression among female caregivers (57.6%) was higher than that of males (34.4%). The higher prevalence of depression among female caregivers could be because females are highly exposed to sexual violence and have a higher rate of experiencing mental health problems such as depression than males.^70^ Also, the diatheses stress model of depression indicated a preponderance of depression among female caregivers, making them more susceptible to depression when facing stressful life events.^71^ Furthermore, biological theories suggest that the genetic susceptibility of depression is also higher among females compared to males.^72^, ^73^ The result suggests more emphasis should be given to female caregivers of cancer patients.

### 4.1 Clinical implications

Our review had important clinical and research implications. Firstly, our review indicated a higher prevalence of depression among caregivers of cancer patients, which suggests the importance of screening depressive symptoms and implementation of evidence‐based interventions. Secondly, further studies should be conducted to examine the possible reasons for a higher prevalence of depression among caregivers of Cancer patients. Thirdly, our sub‐group analysis shown a difference in the prevalence of depression across male and female caregivers, however further studies are essential to strengthen our findings as we found only eight studies which reported the prevalence of depression for male and females. Finally, future systematic reviews and primary studies examining determinants of depression among caregivers of cancer patients are vital for a better understanding of the epidemiology of depression.

### 4.2 Study limitations

The following are the limitations of our review, which need to be considered when inferring our findings. First, all the included studies were only from middle and high‐income countries, which lacks representativeness of low‐income countries. Second, we have included only articles published in English, which might introduce selection bias. Third, it is essential to note the difference between depression assessment tools across the studies could also be a potential limitation. Lastly, most studies included in the current review employed a cross‐sectional design, which has the limitation of providing evidence of a temporal relationship between exposure and outcome.

## 5 CONCLUSION

Our review found that, globally around two in five cancer patient caregivers screened positive for depression, which needs due attention. Early screening of depressive symptoms and targeted psychosocial intervention for caregivers of Cancer patients is highly recommended. Also, expanding on other evidence‐based practices related to addressing depression among caregivers of cancer patients would be beneficial. Further studies should be conducted to examine the possible reasons for a higher prevalence of depression among caregivers of Cancer patients.

## AUTHOR CONTRIBUTIONS

The author Asres Bedaso performed the search, quality appraisal, data extraction, analyses, and writing the draft of the initial manuscript. Bereket Duko and Getiye Dejenu participated in quality appraisal, and data extraction. Getiye Dejenu contributed to the consensus. All authors participated in revising the draft manuscript, and approved the final manuscript.

## CONFLICT OF INTEREST

The authors declare that there is no competing interest.

## Supporting information

Supporting Information S1

## Data Availability

All data generated or analysed during this study are included in this article.
